# MEK5 overexpression is associated with the occurrence and development of colorectal cancer

**DOI:** 10.1186/s12885-016-2327-9

**Published:** 2016-05-09

**Authors:** Dechang Diao, Lei Wang, Jin Wan, Zhiqiang Chen, Junsheng Peng, Huanliang Liu, Xinlin Chen, Wei Wang, Liaonan Zou

**Affiliations:** Department of Gastrointestinal Surgery, Guangdong Provincal Hospital of Traditional Chinese Medicine, Guangdong, 510120 China; Institute of Gastroenterology, Sun Yat-Sen University, Guangzhou, 510655 China; Department of Gastrointestinal Surgery, the Sixth Affiliated Hospital, Sun Yat-sen University, Guangzhou, Guangdong 510655 China; Key Laboratory of Tropical Disease Control (Sun Yat-sen University), Ministry of Education, Guangzhou, Guangdong, 510080 China; Department of Preventive Medicine and Medical Statistics, College of Fundamental Medical Science, Guangzhou University of Traditional Chinese Medicine, Guangdong, 510006 China

**Keywords:** MEK5, Colorectal cancer, Univariate analyses, RNA interference, Tumor growth

## Abstract

**Background:**

Mitogen/extracellular signal-regulated kinase kinase-5 (MEK5) has been confirmed to play a pivotal role in tumor carcinogenesis and progression. However, few studies have investigated the role of MEK5 in colorectal cancer (CRC).

**Methods:**

MEK5 expression was determined by immunohistochemistry (IHC) in tissue microarrays (TMAs) containing 2 groups of tissues, and western blotting was used to confirm MEK5 expression in 8 cases of primary CRC tissues and paired normal mucosa. RNA interference was used to verify the biological function of MEK5 gene in the development of CRC.

**Results:**

IHC revealed the expression of MEK5 was higher in tumor tissues (38.1 %), compared with adjacent normal tissue (8.3 %). Western blot showed that, MEK5 expression was upregulated in CRC tumor tissues compared with normal tissue. Analysis of clinical pathology parameters indicated MEK5 overexpression was significantly correlated with the depth of invasion, lymph node metastasis, distant metastasis and histological grade. Survival analysis revealed that MEK5 overexpression negatively correlated with cancer-free survival (hazard ratio 1.64, *P* = 0.017). RNA interference-mediated knockdown of MEK5 in SW480 colon cancer cells decreased their proliferation, division, migration and invasiveness in vitro and slowed down tumors growth in mice engrafted with the cells.

**Conclusion:**

MEK5 plays an important role in CRC progression and may be a potential molecular target for the treatment of CRC.

## Background

Colorectal cancer (CRC) is a common malignant disease and remains one of the leading causes of cancer mortality worldwide [1]. With the development of China’s economy, the incidence of CRC in China is increasing and now causes a substantial cancer burden in China, particularly in the more developed areas such as Guangdong and Shanghai [2–4]. The carcinogenesis of CRC is often a multistep process and possibly consequent of a complex interaction between multiple factors, both endogenous and environmental stressors [5]. The environmental stressors such as drinking and smoking could lead to activation of many critical molecular pathways, such as mitogen-activated protein kinases (MAPKs) [6], and the Wnt/Wingless signaling pathway [7], eliciting a variety of biological responses.

MAP kinase kinases (MEKs/MAPKKs) represent a family of protein kinases upstream of the MAP kinases, which play an important role in cell proliferation and apoptosis [8]. Mitogen/extracellular signal regulated kinase kinase-5 (MEK5), a key kinase of the MEK5-ERK5 pathway, in turn specifically phosphorylates and activates extracellular signal-regulated kinase-5 (ERK5) [9], which directly phosphorylates and activates several transcription factors including c-Myc, Sap-1, c-Fos, Fra-1, and myocyte enhancer factor family members [10, 11], eliciting a variety of biological responses to extracellular signals that include cytokines, growth factors, and various stress stimuli [12]. The MEK5 cDNA encodes a 444-amino acid protein, which displays approximately 40 % identity to known MEKs [13]. The alternative splicing of the mRNA produces two isoforms with different N-termini, MEK5α (50 kDa) and MEK5β (40 kDa) [14]. The expression of the MEK5β protein is greater than that of MEK5α in terminally differentiated tissues, while MEK5α expression is greater in mitotically active tissues such as the liver. MEK5α directly stimulates ERK5 kinase activity, whereas MEK5β plays a kinase-dead dominant-negative role that suppresses ERK5 signaling [15]. A growing number of studies have shown that overexpression of MEK5α is associated with tumorgenesis and malignancies [16, 17] and the expression ratio of MEK5α to MEK5β is higher in cancer cell lines, while overexpression of MEK5β inhibits serum-induced DNA synthesis [17]. Therefore, alternative splicing of MEK5α and MEK5β may play a pivotal role in ERK5 activation and subsequent carcinogenesis. There are many studies suggesting that MEK5 plays a critical role in cancer occurrence and development, such as prostate cancer [18], breast cancer [19], hepatocellular cancer [20] and lung cancer [21].

We have previously shown the -163 T > C polymorphism in the MEK5 promoter might affect the risk of developing CRC, and further research indicated that the possible mechanism of action might be the effect of -163 T > C variation on the MEK5 expression [22]. Recently, we found that expression of the phosphorylated MEK5 protein was associated with TNM staging of colorectal cancer [23]. In this study, we further investigated the biological role of MEK5 in CRC. We analyzed the relationship between the MEK5 expression and clinicopathological parameters of colorectal carcinoma and assessed the prognostic value of MEK5 in colorectal carcinoma in a large number of patients. Furthermore, we silenced the MEK5 expression in colon cancer cell line SW480 and evaluated the influence of MEK5 on the biological behaviors of colon cancer cells.

## Methods

### Patients and tissue specimens

In this study, immunohistochemstry analysis was conducted on two groups of paraffin-embedded samples. The first group included 24 normal colorectal mucosa, 24 adenomas and 84 primary colorectal adenocarcinomas, which were randomly collected from archival tissues surgically removed at the Sixth Affiliated Hospital of Sun Yat-sen University, between 2007 and 2010. All of these samples were pathologically confirmed. The second group included 342 archival tissues specimens of CRC, which were histologically and clinically diagnosed, from the First Affiliated Hospital of Sun Yat-sen University, between January 2000 and November 2006. The cases selected were based on the following criteria: a distinctive pathological diagnosis of CRC, having undergone primary and curative resection for CRC, availability of resection tissue, availability of follow-up data, and having not received preoperative anticancer treatment. These CRC cases included 185 (54.1 %) men and 157 (45.9 %) women, with a mean age of 59.6 years. The average follow-up time was 71.5 months, and a total of 102 (30.4 %) patients died during the follow-up period. Patients whose cause of death remained unknown were excluded from our study. Tumor grades were defined in accordance with the criteria of the World Health Organization (WHO) (2000). The pathological TNM status of all CRC was defined according to the criteria of the sixth edition of the TNM classification of the International Union Against Cancer (2002). In addition, eight pairs of fresh CRC tissue specimens and normal adjacent colorectal mucosa specimens were obtained from patients with CRC who underwent surgical tissue resection at the Sixth Affiliated Hospital of Sun Yat-sen University during 2010. All of the CRC samples selected were the samples that contained at least 70 % carcinoma tissues in the whole tissue samples with the help of frozen section examination. Our study was approved by Clinical Ethics Review Committee at the Sixth Affiliated Hospital of Sun Yat-sen University (Guangzhou, China), and written informed consent was obtained from all the patients.

### Tissue microarray (TMA)

After pathological review, the representative tumor area in the paraffin block was selected for creation of a tissue microarray (TMA). Two cylinders 1.0 millimeter in diameter were taken from each paraffin block of histologically confirmed specimens to construct the TMAs using Tissue Array (ALPHELYS, MINIPORE). Specifically, the tissue cylinders were taken from the selected region of each donor tissue block and deposited into a recipient block. Then H&E staining was performed on the recipient blocks to verify the adequacy of the tumor, adenomas, and normal tissues.

### Immunohistochemistry analysis

MEK5 expression was examined in the two sets of tissue microarrays by IHC. The expression in normal mucosa, adenoma, and carcinoma was compared, and the potential relationship between MEK5 expression with clinicopathological features and prognosis of adenocarcinomas was also assessed.

The TMAs were sectioned at 4 μm intervals, deparaffinized in xylene, and rehydrated with graded alcohols. The TMAs were then immersed in 3 % hydrogen peroxide for 10 min to block endogenous peroxidase activity, and antigen-retrieved by pressure cooking for 3 min in citrate buffer (pH = 6). The sections were then incubated with polyclonal antibody MEK5 (Rabbit polycolonal antibody, 1:200, Santa Cruz, H-94: sc-10795), at 4 °C overnight. The sections were sequentially incubated with secondary antibody for 30 min at room temperature and stained with DAB. Finally, the sections were counterstained with hematoxylin, dehydrated, and mounted. For negative controls, blocking solution was added instead of the primary antibody. All slides were independently assessed by two pathologists, who were blinded to the cases.

### IHC evaluation

**Fig. 1:**
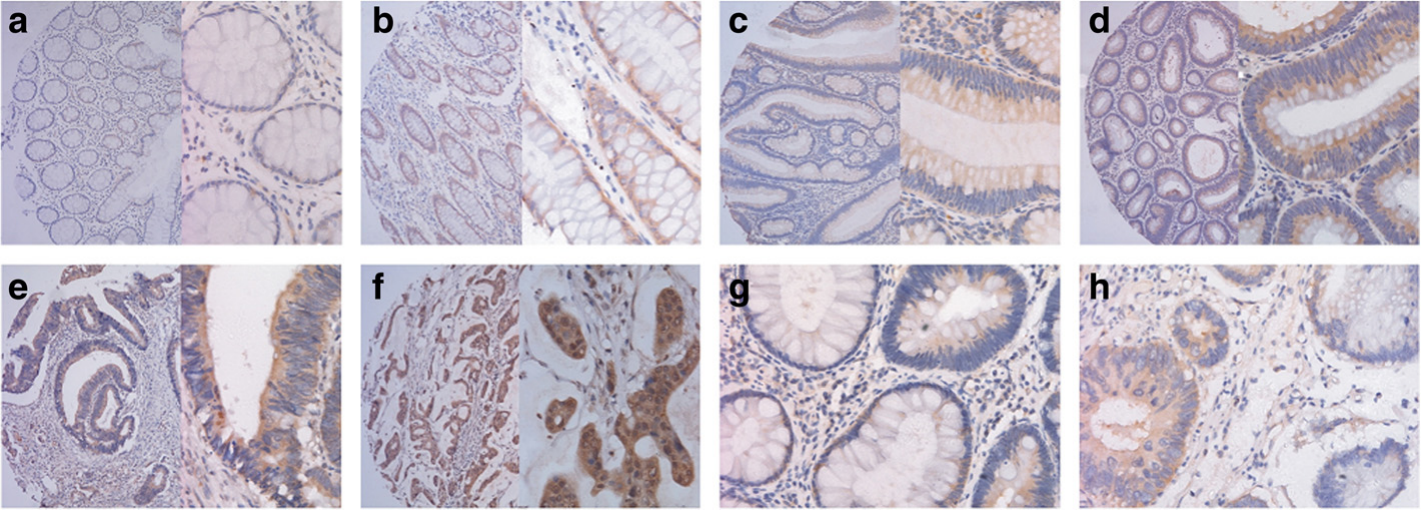
Immunohistochemical staining of MEK5 protein in normal colorectal mucosa, colorectal adenoma, and CRC. (*Left panels*, ×100, right panels, ×400). (**a**) Weak MEK5 expression in normal colorectal mucosa; (**b**) moderate MEK5 expression in adjacent normal colorectal mucosa; (**c**) weak MEK5 expression in colorectal adenoma; (**d**) moderate MEK5 expression in colorectal adenoma; (**e**) weak MEK5 expression in well differentiated CRC; (**f**) strong MEK5 expression in poorly differentiated CRC (mucinous adenocarcinoma). The fig. (**g** and **h**) show the MEK5 expression in the occurrence of CRC: Elevated MEK5 expression in the atypical hyperplasia and tumor cells of CRC tissue compared with those of adjacent normal mucosa

The MEK5 expressions were evaluated semiquantitatively according to the method described by Mehta et al.[8]. The cell was stained mainly in cytoplasm, and the intensity staining was classified as 0 negative; + weak; ++ moderate; +++ intense. For the study, tumors classified as 0 or + were considered to have normal expression and tumors classified as ++ or +++ were considered to have overexpression (Fig. 1). All samples were anonymized and independently scored by two trained pathologists. Scoring was performed blindly and without knowledge of the eventual clinical parameters. When differences between inter-observers occurred, the slides in question were jointly reexamined by two investigators.

### Cell line and cell culture

The human colon cell line SW480 was purchased from the Type Culture Collection of Chinese Academy of Sciences (Shanghai, China). The cancer cells were maintained in RPMI 1640 medium (Hyclone, USA) supplemented with 10 % fetal bovine serum (FBS) and 100 units/ml penicillin, and 100 mg/ml streptomycin in flasks at 37 °C in an environment with 5 % CO2. Stock culture of the cell line was routinely sub-cultured at least once a week with the medium changed every 2–3 days.

### SiRNA mediated MEK5 knockdown

To knockdown MEK5 expression, lentiviral-MEK5-siRNA vectors targeting human MEK5 and Nonsilencing MEK5 control vector contained the sequences encoding green fluorescent protein (GFP) were designed and constructed by Cyagen Biosciences Inc.. The shRNA sequence was designed to target MEK5 as follows: MEK5sh1, 5′-GAGAACCAGGTGCTGGTAATT-3′; MEK5sh2, 5′- GCCCTCCAATATGCTAGTAAA-3′; MEK5sh3, 5′- CCGTTCATCGTGCAGTTCAAT -3′. SW480 cells were seeded in six-well plates at a density of 5 × 10^5^ cells/well and grown overnight until 70–80 % confluence was achieved to obtain maximum transfection efficiency. Transfection of the lentivirus for SW480 cells were performed with Lipofectamine 2000 (Invitrogen, Carlsbad, CA) according to the manufacturer’s instructions followed by puromycin selection (1 μg/mL) for 6 days. Cells were divided into three groups as follows: the knockdown (KD) cells were transfected with MEK5 shRNA lentivirus (MOI 20); the negative control (NC) cells were transfected with empty lentivirus (MOI 20) and the blank control (BC) cells were not transfected. The silencing efficiency of MEK5 was assayed by real-time quantitative-PCR (qPCR) and western blot at 48 h post-transfection.

### Western blot analysis

Protein samples (20 μg) were separated by 10 % acrylamide gel using a Bio-Rad Mini-Protein III system (100 V for 2 h) and then transferred to PVDF membranes in 200 mA for 50 min in transfer buffer. The membranes were blocked for 90 min with 5 % skimmed milk powder in 0.05 % TBS-T at room temperature. The monoclonal antibody against MEK5 was purchased from BD Transduction Laboratories (San Diego, CA, USA), and the monoclonal antibody against β-actin was purchased from Santa Cruz Biotechnology. The membranes were then incubated overnight at 4 °C with primary antibodies in 2 % BSA dissolved in TBS-T (1:500 dilution), and the proteins were detected with a Phototope-horseradish peroxidase Western blot detection kit (Cell Signaling Technology, Inc). Protein expression levels were normalized to that of β-actin by calculating the relative expression levels.

### RNA extraction and qRT-PCR

Total RNA extraction was carried out using Trizol reagent (Invitrogen) according to the manufacturer’s instruction. Two microgram of total RNA was subjected to reverse Transcription (RT) using Verso cDNA Ki (Thermo Scientific). Real-time quantitative PCR was conducted by Platinum SYBR Green qPCR SuperMix UDG with ROX kit (Invitrogen) in 20 μl and ABI 7300 real-time PCR thermal cycle instrument (ABI, USA) according to the supplied protocol. The primers for MEK5 were: (F, CTTTAATGCCTCTCCAGCTTCT; R, CCATCATTGAACTGCACGAT). The relative expression levels were normalized to expression of endogenous GAPDH. (Primers: F, GGGAAACTGTGGCGTGAT; R, GAGTGGGTGTCGCTGTTGA).

### Cell proliferation assay

Cell Counting Kit-8 (CCK-8; Dojindo) was used in cell proliferation assay. 5 × 10^3^ cells/well viable cells were seeded in 96-well tissue culture plates in a final volume of 100 μl. At time points of day 0, day 2, day 3 and day 4, a plate was subjected to assay by adding 10 μl of CCK-8 solution to each well, and the plate was further incubated at 37 °C for 4 h, and then the absorbance at 450 nm was calculated. The experiment was performed in eight replicates.

### Cell cycle analysis

Following transfection 48 h later, 1 × 10^6^ cells were collected, washed in PBS, fixed in 70 % ethanol, and kept at 4 °C overnight. The cells were resuspended to a concentration of 1 × 10^6^ cells/ml in PBS and incubated with 100 μg/ml RNase A and 50 mg/ml propidium iodide (PI) at 4 °C for 30 min. The total cellular DNA content was analyzed by flow cytometry (Becton Dickinson, San Jose, CA).

### Cell migration assay

Cell migration was evaluated by scratch wound assay [24]. In brief, SW480 cells were plated in 6-well plate at concentration of 10^6^/well and cultured overnight to yield confluent monolayer. Next, the cells were treated with 10 mg/ml mitomycin for 1 h to inhibit proliferation, followed by wounding with 10 ml pipette tip. Remaining cells were washed twice and then cultured with serum free RPMI-1640 medium. Photographic images were taken from each well at 0 h, 6 h, 24 h and 48 h. The distance that cells migrated through the area created by scratching was caculated by measuring the wound width at the above times and subtracting it from the wound width at the start. The values obtained were then expressed as the rate of wound healing. The experiments were performed at least in triplicate.

### Cell invasion assay

Cell invasion was evaluated by transwell matrigel invasion assay using BD Biocoat Matrigel invasion chambers (BD Biosciences, USA). Briefly, 500 μl of the cell suspended in serum free RPMI-1640 medium at a concentration of 1 × 10^5^ cells was added to the upper compartment, while the lower compartment contained 750 μl medium with EGF (15 ng/mL) additionally. After 24 h of incubation, chambers were rinsed and the Matrigel matrix and noninvading cells on the upper surface of the membrane were removed using moistened cotton swabs. Afterwards, cells on the lower surface were fixed with methanol and stainedwith 0.1 % toluidine blue. Membranes were cut out and evaluated under microscopic by placing on microscope slides.

### In vivo tumor model

Six 4-week-old athymic BALB/C nude mice (male, 14–16 g) were purchased from the Laboratory Animal Center of Southern Medical University (Guangzhou, China). The animals were housed in SPF under identical conditions and allowed free access to a standard diet and tap water with 12-h light and dark cycles, under an experimental protocol approved by the Institutional Animal Care and Use Committee (IACUC) of Guangdong Provincal Hospital of Traditional Chinese Medicine. All operations were performed under clean conditions. KD cells (5 × 10^6^ in 0.1 ml of PBS) were injected subcutaneously into the left dorsal flank of each mice, while the same number of NC cells injected subcutaneously into the right dorsal flank. Tumor mass volume, which was calculated as (length/2) × (width/2), was measured every two days from day 7 to day 21. On day 21 the NC tumors all began to fester therefore the six mice were sacrificed and all tumors were harvested. Then MEK5 protein expression in tumors was detected by western blot analysis as described above. The experimental procedures were done in accordance with the ARRIVE guidelines.

### Statistical analysis

All of the experimental data were analyzed by using the statistical software SPSS 17.0. The statistical methods used included chi-square tests and paired sample’s t tests. The chi-square test and Fisher’s exact test were used to examine the association between MEK5 expression and various clinicopathological parameters. Univariate analyses were conducted using the Kaplan-Meier method, and statistical significance between survival curves was assessed by the log-rank test. Univariate Cox proportional hazards regressions were applied to estimate the individual hazard ratios (HR) for disease-free survival (DFS) and overall survival (OS). The variables that were significant in the univariate analysis (*P* < 0.05) were then included into the multivariate analysis. The HR with 95 % confidence interval (CI) was measured to estimate the hazard risk of individual factors. Significant differences between the groups were determined using the unpaired Student’s *t*-test. All tests were two-sided, and a *p*-value less than 0.05 was considered statistically significant.

## Results

### MEK5 expression in CRC tissue and normal colorectal mucosa samples

Immunostaining of MEK5 in CRC tissues and normal mucosa was detected as brown-yellow granules in the cytoplasm (Fig. 1). In the first group object of this study, the MEK5 was overexpressed in 38.1 % of CRC tissues (32 out of 84); compared with 20.8 % of colorectal adenoma (5 out of 24) and 8.3 % of normal tissues (2 out of 24) (Fig. 1). Statistical analysis indicated that MEK5 was gradually up-regulated from normal mucosa to adenomas, and to tumor tissues (*P* = 0.011; Table 1). Furthermore, in some sections of colorectal adenomas and at the junctions of tumor and normal mucosa, we found that the MEK5 expression level was notably correlated with progression of CRC. MEK5 expression was normal in normal colorectal mucosa and higher in the adjacent atypical hyperplasia of the mucosa (Fig. 1-g, h).

**Table 1:** MEK5 expression in normal mucosa, adenoma and CRC tissues

Tissue type		MEK5 expression		*χ* ^2^	
	All cases	Normal (%)	Over (%)		*P* value
				9.01^a^	0.011^a^
Normal	24	22(91.7 %)	2(8.3 %)	1.51^b^	0.220^b^
Adenoma	24	19(79.2 %)	5(20.8 %)	2.27^c^	0.116^c^
Carcinoma	84	52(61.9 %)	32(38.1 %)	7.67^d^	0.006^d^

^a^The *χ* ^2^ and *P* value of the three groups; ^b^the *χ* ^2^ and *P* value of normal colorectal mucosa V.S. colorectal adenoma; ^c^the *χ* ^2^ and *P* value of colorectal adenoma V.S. CRC; ^d^the *χ* ^2^ and *P* value of normal colorectal mucosa V.S. CRC

Normal, negative or weak; over, moderate or intense

**Fig. 2:**
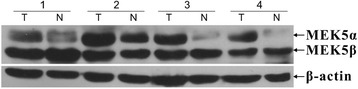
Western blot analysis of MEK5 protein expression. Western blot analysis of MEK5 proteins expressed in eight pairs represents colorectal tumor tissues (T) and their matched adjacent non tumor tissues (N). Expression level was normalized with β-actin

To confirm the expression levels of MEK5 seen by immunostaining in the specimens from our TMA, we examined the expression of MEK5 protein by western blot analysis in 8 randomly selected pairs of CRC tissues and their matched not-tumor colorectal tissues. In 5 of 8 (62.5 %) CRC patients, the total MEK5 protein was up-regulated in tumor tissues compared with their adjacent nontumor colorectal mucosa; furthermore, the ratio of MEK5α to MEKβ was higher in all of the CRC tissues than in their adjacent normal colorectal mucosa (Fig. 2).

### Correlations of MEK5 protein expression and clinicopathologic parameters

In our study, overexpression of MEK5 protein was observed in 109 of the 342 CRC tissues (31.9 %) in the second group samples. The relationship between immunohistochemical MEK5 expression in CRC tissues and various clinicopathologic characteristics is shown in Table 2. The results demonstrated that high expression of MEK5 was associated with depth of invasion (*P* = 0.001), lymph node metastasis (*P* = 0.001), distant metastasis (*P* = 0.026), TNM stage (*P* < 0.001) and differentiation grade (*P* = 0.002). There was no significant association between MEK5 expression and other clinicopathologic features, including age, sex, tumor location, pathology type and serosal invasion.

**Table 2:** Correlation between MEK5 expression and clinicopathologic characteristics

		MEK5 expression		
	All cases	Low (%)	High (%)	*P* value
Sex				0.648
Female	157	105(66.9)	52(33.1)	
Male	185	128(69.2)	57(30.8)	
Age at diagnosis (years)				0.697
< 60	159	110(69.2)	49(30.8)	
≥ 60	183	123(67.2)	60(32.8)	
Tumor location				0.815
Rectum	160	108(67.5)	52(32.5)	
Colon	182	125(68.7)	57(31.3)	
pT (invasion depth)				0.001
T1	5	5(100)	0(0)	
T2	57	50(87.7)	7(12.3)	
T3	242	157(64.9)	85(35.1)	
T4	38	21(55.3)	17(44.7)	
pN (lymph node metastasis)				0.001
N0	205	155(75.6)	50(24.4)	
N1	87	47(54)	40(46)	
N2	50	31(62)	19(38)	
pM (distant metastasis)				0.026
M0	312	218(69.9)	94(30.1)	
M1	30	15(50)	15(50)	
TNM stage				0.000
I	48	45(93.8)	3(6.3)	
II	147	104(70.7)	43(29.3)	
III	117	69(59)	48(41)	
IV	30	15(50)	15(50)	
Differentiation grade				0.002
Well	28	25(89.3)	3(10.7)	
Moderate	284	194(68.3)	90(31.7)	
Poorly	30	14(46.7)	16(53.3)	
Pathology type				0.812
Villous adenocarcinoma	21	16(76.2)	5(23.8)	
Tubular adenocarcinoma	277	187(67.5)	90(32.5)	
Mucinous adenocarcinoma	29	19(65.5)	10(34.5)	
Others	15	11(73.3)	4(26.7)	
Serosal invasion				0.227
Yes	73	54(74)	19(26)	
No	269	179(66.5)	90(33.5)	

Low, negative or weak; high, moderate or intense

### Survival analysis

**Fig. 3:**
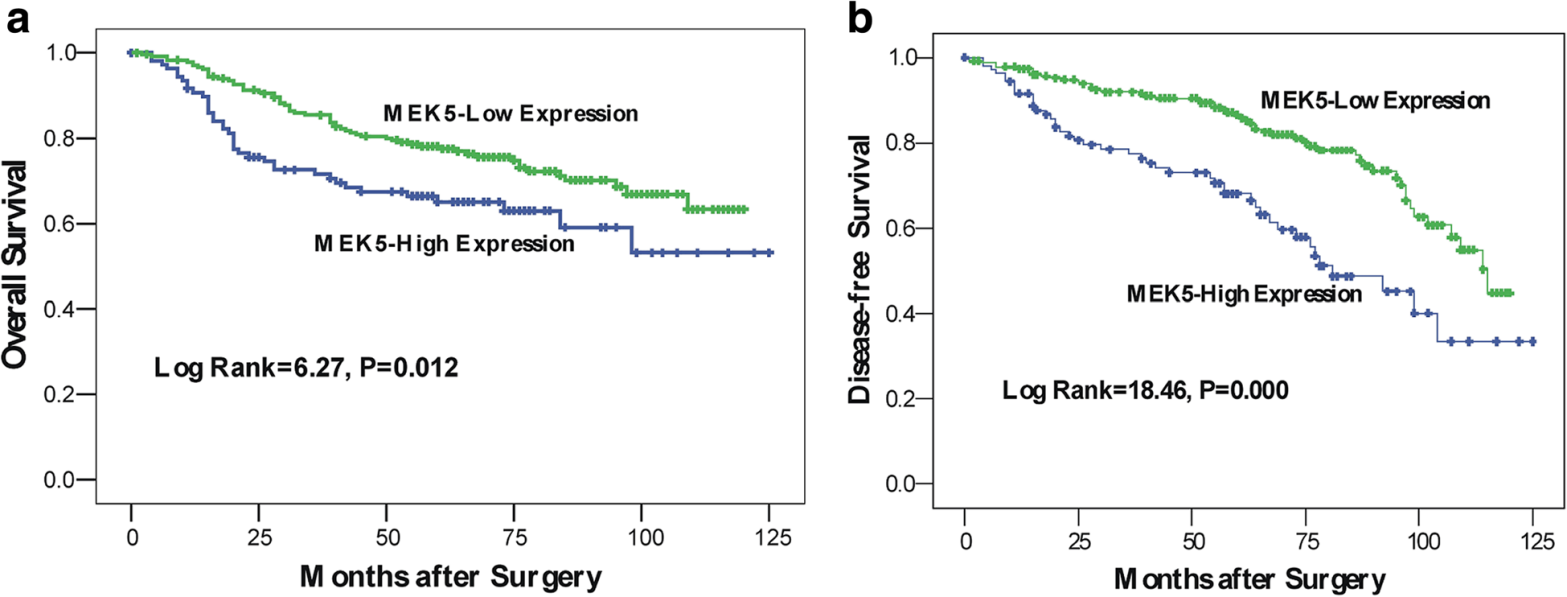
Survival analysis of primary CRC patients (*n* = 342). Kaplan-Meier estimates of the DFS (**a**) and OS (**b**) according to MEK5 expression in 342 patients. The DFS and OS were significantly lower in patients with MEK5-high expression when compared with patients who had low MEK5 expression. *P* values were calculated using the log-rank test

The mean patient follow-up time was 71.5 months and the 5-year OS rate of the 342 patients with primary colorectal cancer was 69.6 %, with 102 deaths observed during the follow-up period. The 5-year DFS rate was 67.8 %. During the time of follow-up, 82 patients (24.5 %) developed distant metastasis or local recurrence. According to the univariate analyses, tumor location, TNM stage and differentiation grade were significantly associated with patients’ overall survival and disease-free survival (*P* < 0.05; Tables 3 and 4). Assessment of CRC patient survival also revealed that overexpression of MEK5 was significantly correlated with short disease-free survival (*P* < 0.001, Table 3 and Fig. 3-a) and poor overall survival (*P* = 0.012, Table 4 and Fig. 3-b).

**Table 3:** Cox proportional hazards model univariate and multivariate analyses of individual parameters for correlations with disease-free survival (342 cases)

	Univariate analysis			Multivariate analysis	
	All cases	Mean survival (months)	*P* value	HR (95 % CI)	*P* value
Sex			0.938		
Female	157	93.5			
Male	185	92.9			
Age at diagnosis (years)			0.754		
< 60	159	91.0			
≥ 60	183	94.7			
Tumor location			0.011	1.850 (1.23–2.78)	0.003
Rectum	160	95.2			
Colon	182	84.2			
TNM stage			0.000	2.70 (1.81–4.01)	0.000
I-II	195	120.7			
III-IV	147	64.6			
Differentiation grade			0.000	1.88(1.07–3.32)	0.029
Well and Moderate	312	96.6			
Poorly	30	61.8			
Serosal invasion			0.230		
Yes	73	98.8			
No	269	92.3			
MEK5 expression			0.000	1.64 (1.09–2.47)	0.017
Normal expression	233	98.2			
Over expression	109	79.3

**Table 4:** Cox proportional hazards model univariate and multivariate analyses of individual parameters for correlations with overall survival (342 cases)

	Univariate analysis			Multivariate analysis	
	All cases	Mean survival (months)	*P* value	HR (95 % CI)	*P* value
Sex			0.988		
Female	157	94.2			
Male	185	91.7			
Age at diagnosis (years)			0.144		
< 60	159	94.9			
≥ 60	183	90.6			
Tumor location			0.023	1.76(1.17–2.67)	0.008
Rectum	160	96.5			
Colon	182	88.8			
TNM stage			0.000	2.75(1.82–4.16)	0.000
I-II	195	106.1			
III-IV	147	73.0			
Differentiation grade			0.001	2.40(1.34–4.28)	0.003
Well and Moderate	312	96.5			
Poorly	30	60.9			
Serosal invasion			0.411		
Yes	73	95.8			
No	269	93.1			
MEK5 expression			0.012	1.51(1.01–2.25)	0.045
Normal expression	233	95.2			
Over expression	109	84.8

In order to address potential confounding among variables examined in the univariate analysis, we conducted multivariate analysis using Cox proportional hazards model for all of the significant variables in the univariate analysis. We found that overexpression of MEK5 was an independent risk factor for poor disease-free survival (HR: 1.64; 95 % CI: 1.09–2.47; *P* = 0.017). Of the other variables, tumor location, TNM stage and differentiation grade were also found to be independent prognostic predictors for disease-free survival (Table 3). On the other hand, MEK5 overexpression, tumor location, TNM stage and differentiation grade were found to be independent prognostic predictors for overall survival (Table 4).

### Knockdown of MEK5 expression in SW480 cells

**Fig. 4:**
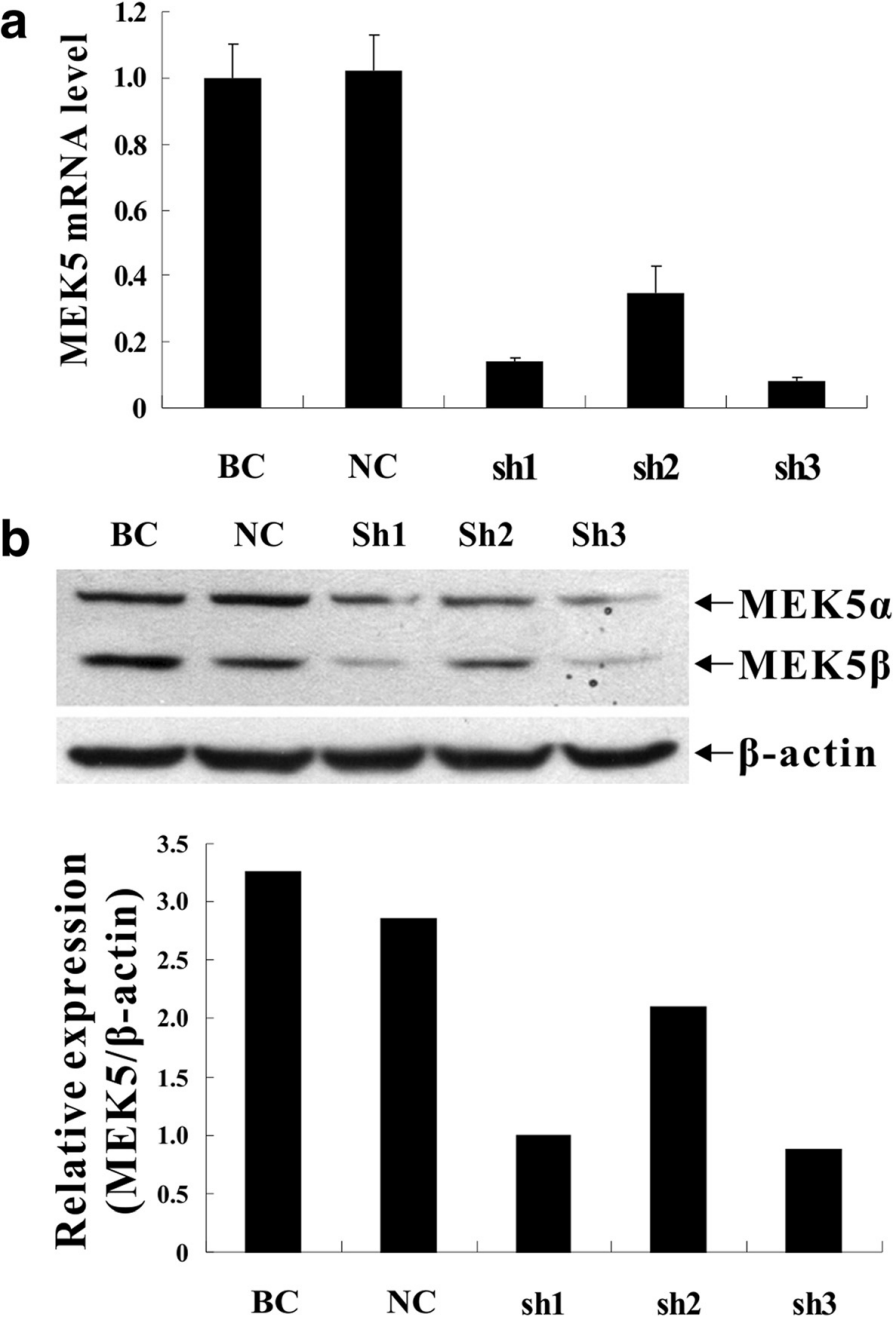
Knockdown of MEK5 gene by MEK5 shRNA lentivirus. **a** qRT-PCR showed a significant decrease of MEK5 mRNA (by 86.3 %) in the sh3 group vs. BC group. **b** Western blot assay demonstrated that, normalized by β-actin, MEK5 protein expression was degraded (by 69.6 %) in the sh3 group vs. BC group. sh1: MEK5sh1; sh2: MEK5sh2; sh3: MEK5sh3; NC, negative control; BC, blank control; KD, knockdown

After 48 h transfection, green fluorescent protein (GFP) expression rates of the KD and NC cells were all more than 80 %, respectively. When compared with the parental NC cells and BC cells, the three lentiviral-MEK5-siRNA vectors transfected cells showed obvious decreases in the mRNA and protein expressions of MEK5. In particular, the silencing efficiency of lentiviral-MEK5-siRNA-3 was the highest, with the reduction of MEK5 mRNA expression by 86.3 % (*P* = 0.025) and protein expression by 69.6 % (Fig. 4) comparing with BC cells. Therefore, the SW480 cells carrying lentiviral-MEK5-siRNA-3, NC cells and BC cells with stable expression were harvested after puromycin selection.

### Effect of MEK5 knockdown on the biological behavior of SW480 Cells in vitro

**Fig. 5:**
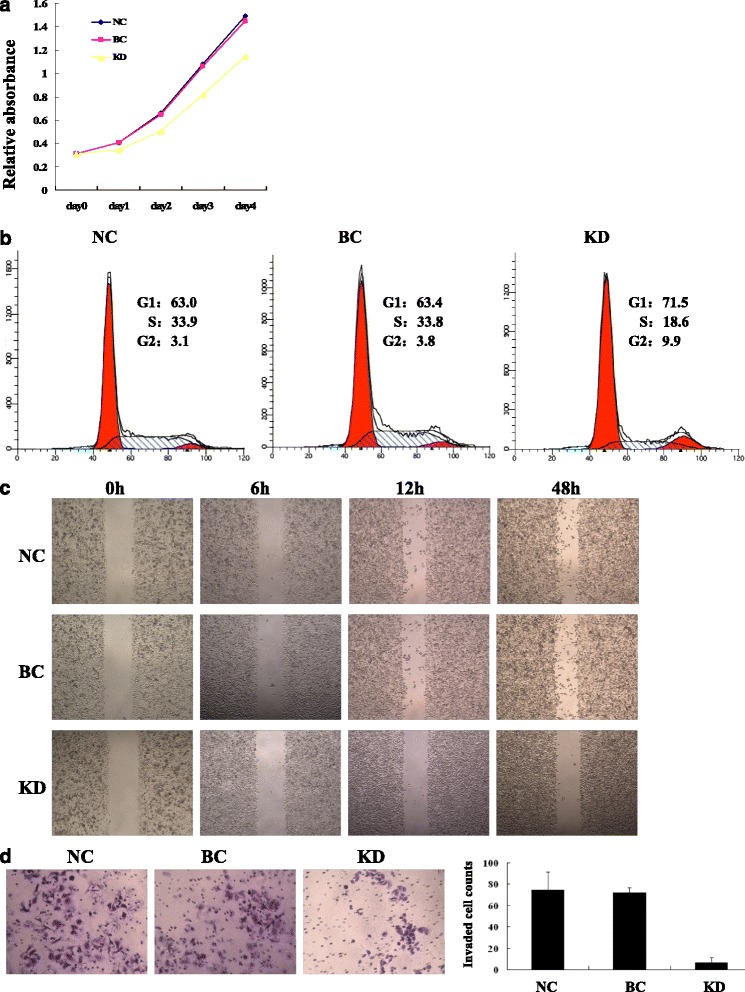
Effects of MEK5 knockdown on proliferation, division, migration and invasiveness of SW480 cell in vitro. The proliferation ability of NC group, BC group and KD group was examined by CCK-8, cell division was examined by flow cytometry, and migrated ability was tested by scratch assay and invasive ability was examined by transwell Matrigel invasion assay. Comparing with NC and BC groups, the proliferation (**a**), division (**b**), migration (**c**) and invasiveness (**d**) of siRNA treated cells (KD group) were significantly decreased. NC, negative control; BC, blank control; KD, knockdown

Cell Counting Kit 8 (CCK-8) assay showed that knockdown of MEK5 expression significantly inhibited the proliferation of SW480 cell, indicating that MEK5 gene expression affects the growth of colon cancer cells (Fig. 5a). The flow cytometery results showed that, in the NC group 63.43 % of cells were in the G1 phase and 32.77 % of the cells were in S of the cell cycle, and in the BC group 63.02 % of cells were in the G1 phase and 33.87 % of the cells were in S of the cell cycle, while in KD group cells accumulated in G1 71.53 % but reduced to 18.61 % in S phase (Fig. 5b). In scratch wound assay, migration ability of KD group was obviously inhibited than that of NC group and BC group (Fig. 5c), indicating silence of MEK5 gene led to a significantly decreased migration ability of SW480 cells. Transwell matrigel invasion assay showed that silencing of MEK5 expression significantly inhibited invasion of SW480 cells in vitro (*P* < 0.01, Fig. 5d).

### In vivo studies of SW480 cells xenograft tumor models in nude mice

**Fig. 6:**
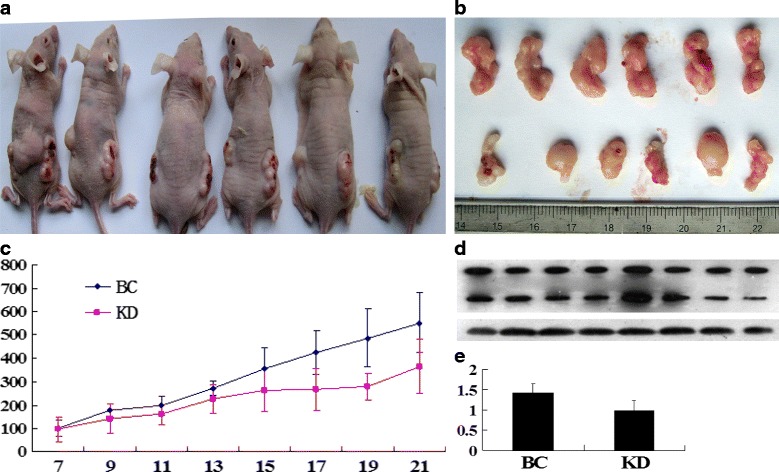
Silencing of MEK5 significantly inhibited cancer growth in vivo. **a** KD cells (5 × 10^6^ in 0.1 ml of PBS) were injected subcutaneously into the left dorsal flank of each BALB/C nude mice, while the same number of BC cells injected subcutaneously into the right dorsal flank. **b** Tumor mass volume was measured every two days from day 7 to day 21. On day 21 the six mice were sacrificed and all tumors were harvested. **c** Silencing of MEK5 could significantly inhibited the cancer growth, when compared with BC cells (*P* < 0.05). **d** Western blot assay showed MEK5 protein expression of the xenograft tumors of KD cells was significantly inhibited comparing with that of NC cells. NC, negative control; KD, knockdown

To further evaluate the role of reduced MEK5 expression on the tumorigenic phenotype and in particular its contribution to in vivo tumor growth. SW480 cells infected with non-silencing shRNA and MEK5 shRNA were injected into 6 mice, (Fig. 6a). The cancer growth curves in nude mice after injection of MEK5 shRNA transfected cells and the control cells are shown in Fig. 6c. The tumor growth speed of the KD cells was obvious slower than that of NC cells (*P* < 0.05). These results demonstrate that in vivo tumor growth was inhibited by shRNA-mediated knockdown of MEK5 expression in colon cancer cells. Western blot assay showed MEK5 protein expression of the xenograft tumors of KD cells was significantly inhibited comparing with that of NC cells (*P* < 0.01, Fig. 6d).

## Discussion

The occurrence and development of CRC is correlated with various molecular and genetic incidents. Recent data have been accumulating to support a key role of MEK5/ERK5 signaling in carcinogenesis [25], and several studies have demonstrated that tumor cells can acquire cancerous capacity by increasing expression of MEK5 to activate ERK5. In metastatic prostate cancer, strong MEK5 expression is correlated with bony metastases, and less favorable prognosis is caused by up-regulated ERK5-induced activator protein-1 (AP-1) activity, a consequent induction of a high level of matrix metallo-protease-9 (MMP-9) and augmented invasive potential [8]. Dudderidge et al. showed that the induction of MEK5/ERK5 signalling was associated with activation of the DNA replication licensing pathway in prostate cancer [26]. Ghosh AK et al. demonstrated that exogenous expression of c-myc promoter-binding protein 1 (MBP-1) induces prostate cancer cell death by down-regulating the expression of MEK5α and up-regulating the level of MEK5β [16]. Hui Song et al. observed that MEK5α might be one of the Stat3-regulated genes and play a critical role in oncogenesis mediated by aberrantly activated Stat3 signaling in breast carcinomatosis and malignancies [27]. Recently, we found that pMEK5 expression was correlated with the staging of CRC and its expression might be helpful for the TNM staging system of CRC [23].

In this study, we examined the expression of MEK5 by IHC in 24 normal colorectal mucosa, 24 adenomas and 84 primary colorectal adenocarcinomas, and found that MEK5 was gradually up-regulated in the development of CRC from normal mucosa, through adenomas, to cancer. Moreover, we found that the MEK5 expression status was notably correlated with progression of CRC in the same pathological section. Elevated MEK5 expression was found in the adjacent atypical hyperplasia of the mucosa compared with those of normal colorectal mucosa. In order to confirm the results seen by immunostaining the specimens from our TMA, we examined the expression of MEK5 protein by western blot in 8 randomly selected pairs of CRC tissues and their matched normal adjacent mucosa. The results demonstrated that the major CRC tissues examined had higher levels of MEK5 expression than adjacent normal mucosa. These findings suggest that up-regulated expression of MEK5 may provide a selective advantage in CRC tumorigenesis. In the immunostaining of MEK5 in the 342 cases of CRC, we found that the expression of MEK5 was positively correlated with clinical stage and differentiation grade. These data suggest that overexpression of MEK5 may facilitate the invasive/metastatic phenotypes of human colorectal cancer.

Another interesting finding was that, in the western blot testing of MEK5 in 8 pairs of CRC tissues, we found the ratios of MEK5α to MEK5β were higher in all CRC tissues than that in adjacent normal mucosa. It is known that MEK5α induces cell proliferation by activating its downstream molecules, whereas MEK5β expression is associated with inhibition of cell growth. This result indicated that the ratio of MEK5α to MEK5β might be more important than the total amount of MEK5 expression in the activation of MEK5/ERK5 signaling and progression of carcinogenesis. Activation of MEK5/ERK5 signal has been demonstrated in cells in response to extracellular signals that include cytokines, growth factors, and various stress stimuli. Alternative splicing of MEK5α and MEK5β plays a significant role in ERK5 activation and subsequently induce carcinogenesis [13]. We hypothesized that, as a result of long-time extracellular stimulation, the MEK5/ERK5 pathway in the colorectal mucosa cells was activated excessively, and eventually induced malignant growth. Targeting MEK5 kinase activity or blocking the MEK5/ERK5 pathway may provide an additional means of inhibiting cell migration associated with CRC progression to metastasis.

MEK5 may have clinical value for prognosis or treatment. Weldon et al. reported that drug-resistant MCF7 cells appeared to have significantly high level of MEK5. In that report, MEK5 contributed to prevention of cell apoptosis and chemotherapeutic resistance [28]. In our study, Kaplan-Meier analysis of the survival curves showed a significantly worse 5-year DFS and 5-year OS rate for patients whose tumors overexpressed MEK5. This suggests that MEK5 protein may be a biomarker for poor prognosis for CRC patients. In the multivariate analysis, the result showed that overexpression of MEK5 was an independent predictor of shortened DFS and poor OS. Therefore, the CRC patients with MEK5 overexpression may require a more powerful adjunctive therapy and intensive follow-up. Whether MEK5 has value clinically as a biomarker for therapeutic approaches in patients with colorectal cancer should be followed up with additional appropriately designed studies.

In order to provide further support that MEK5 contributes to the development and progression of colon cancer, the colon cancer cell line SW480 was employed for function experiment. We effectively down-regulated MEK5 expression in SW480 cells by lentiviral-MEK5-shRNA in vitro, and our data indicated that proliferation, cell cycle progression, migration and invasiveness of stable transfected cells were significantly decreased. Finally, we showed that down-regulation of MEK5 in SW480 cells resulted in slower tumor growth in vivo. Taken together, the function experiments further confirmed that down-regulation of MEK5 could inhibit the proliferation and aggressiveness of colon cancer cell line in vitro, and negatively affected development of tumors in vivo, which were consistent with our data from the immunohistochemical and western blot analysis using the clinical CRC samples. In the future study, a larger sampler size is needed to validate this result.

## Conclusion

The overexpression of MEK5 could be used as an effective additional tool in identifying those CRC patients at increased risk of tumor invasiveness, metastasis, or differentiation grade, and knock down of MEK5 led to significantly inhibiting the malignant phenotype of colon cancer cells in vitro an vivo. Moreover, MEK5 could be an encouraging novel molecular biomarker to predict the prognosis of CRC patients and may be a potential molecular target for the treatment of CRC.

## Abbreviations

BC: blank control
CI: confidence intervals
CRC: colorectal cancer
DFS: disease-free survival
ERK5: extracellular signal-regulated kinase-5
HR: hazards ratio
IHC: immunohistochemistry
KD: knockdown
MAPK: mitogen-activated protein kinase
MEK5: mitogen/extracellular signal regulated kinase kinase-5
NC: negative control
OS: overall survival
TMA: tissue microarray
